# Hypometabolic Responses to Chronic Hypoxia: A Potential Role for Membrane Lipids

**DOI:** 10.3390/metabo11080503

**Published:** 2021-07-31

**Authors:** Elie Farhat, Jean-Michel Weber

**Affiliations:** Biology Department, University of Ottawa, Ottawa, ON K1N 6N5, Canada; efarh086@uottawa.ca

**Keywords:** metabolic suppression, hypometabolism, hypoxia tolerance, low oxygen stress, membrane remodeling, cholesterol, phospholipids, fatty acids, Na^+^/K^+^-ATPase, energy metabolism, mitochondrial respiration

## Abstract

Metabolic suppression is an essential strategy to cope with chronic hypoxia. This review examines the physiological processes used to survive in low oxygen environments. It proposes a novel mechanism–the *remodeling of membrane lipids*–to suppress ATP use and production. Temperature (homeoviscous adaptation), diet (natural doping in migrant birds) and body mass (membrane pacemaker of metabolism) have an impact on the lipid composition of membranes, which, in turn, modulates metabolic capacity. Vertebrate champions of hypoxia tolerance show extensive changes in membrane lipids upon in vivo exposure to low oxygen. These changes and those observed in hibernating mammals can promote the downregulation of ion pumps (major ATP consumers), ion channels, mitochondrial respiration capacity (state 3, proton leak, cytochrome c oxidase), and energy metabolism (β-oxidation and glycolysis). A common membrane signal regulating the joint inhibition of ion pumps and channels could be an exquisite way to preserve the balance between ATP supply and demand in hypometabolic states. Membrane remodeling together with more traditional mechanisms could work in concert to cause metabolic suppression.

## 1. Introduction

Hypoxia is a ubiquitous state of low oxygen common to many aquatic and terrestrial environments. It occurs in warm waters where O_2_ solubility is low, in ice-covered lakes and streams where exchange through the surface is restricted, at high altitude, and in deep underground burrows [1,2,3]. Global warming is exacerbating the problem, particularly in oceans, where oxygen minimum “dead” zones are expanding rapidly [4]. Except for a few unusually tolerant species, hypoxia is lethal to most animals as they eventually fail to match ATP supply with demand [5]. Champions of hypoxia tolerance like some cyprinid fish, freshwater turtles and African mole rats can easily withstand several weeks of low O_2_ by suppressing their metabolic rate [6,7]. This critical response can only be achieved through the parallel downregulation of ATP-consuming processes [5] and energy metabolism [8,9,10]. The simultaneous reduction in ATP supply and demand is realized via post-translational and post-transcriptional modifications involving phosphorylation/dephosphorylation reactions, association of enzymes with subcellular structures [11], activation of 5′-AMP-activated protein kinase (AMPK) [12], and epigenetic mechanisms [13]. It has been proposed that altering the composition of membrane lipids could also be used to promote hypometabolism [14,15]. The membrane changes discovered in these studies can contribute to metabolic suppression by reducing the activity of integral proteins. Extensive work on artificial or manipulated natural membranes has characterized a clear functional link between the lipid composition of membranes and the activity of embedded proteins [16,17,18,19,20,21,22]. Recent experiments have demonstrated that goldfish [14] and naked mole-rats (NMR) [15] exposed to chronic hypoxia alter membrane lipids in tissue-specific ways that can promote metabolic suppression.

The goal of this review is to examine current information on the physiological mechanisms used by tolerant species to suppress metabolic rate in chronic hypoxia. Here, we define chronic hypoxia as exposure to low environmental O_2_ for a minimum of one week. Particular emphasis is placed on how chronic hypoxia alters: (i) membrane responses and how changes in their lipid constituents could modulate ion pumps (ATP use) and channels, (ii) key enzymes of energy metabolism (ATP supply), and (iii) mitochondrial respiration. The functional contributions of these 3 levels of regulation to the orchestration of metabolic suppression are addressed. The roles of AMPK and epigenetics are not further discussed here because they have been extensively reviewed elsewhere [12,13].

## 2. Membrane Lipids: Response to Environmental Stress and How They Affect Metabolism, Ion Pumps and Channels

To remain functional in variable environments, membranes modify their chemical structure in multiple ways. This section deals with the effects of chronic hypoxia on the lipid composition of membranes. It starts by examining previous work on the well characterized effects of temperature, toxins, diet, and phylogeny to illustrate how hypoxia-driven membrane remodeling can be used to suppress metabolism.

### 2.1. Temperature and Toxins

Membrane fluidity varies with temperature [23], but most animals manage to keep it constant by altering their lipid constituents–phospholipids and cholesterol–through a mechanism known as homeoviscous adaptation [24,25]. This response is most common in ectotherms [24,26,27], but has also been reported in isolated mammalian cells [26]. Animals counteract the effects of increasing temperature on membrane fluidity by decreasing the degree of unsaturation and/or increasing the fatty acid chain length of phospholipids [23]. Because cholesterol affects the interactions between phospholipids, changes in its abundance are also used to stabilize membrane fluidity and cope with a variety of environmental stresses. Cholesterol promotes an “intermediate state” in phospholipids that causes an increase in fluidity below and a decrease in fluidity above the liquid-gel phase transition temperature [27]. It also interacts with the polar head groups of phospholipids to decrease membrane permeability [28,29]. Interestingly, homeoviscous adaptation can even occur in response to environmental pollutants. For example, goldfish chronically exposed to the membrane fluidizer PCB-153 can use changes in cholesterol abundance to counteract the effects of the toxin and maintain constant fluidity [30].

### 2.2. Diet

Membranes are affected by the lipid composition of the diet in various animal groups including fish [31], birds [32,33] and mammals [34]. Some species use this mechanism strategically to prepare for hibernation [35] or long-distance migration [36]. The likelihood of golden-mantled ground squirrels to enter and survive hibernation is greatly increased when they switch from a high polyunsaturated fatty acid (PUFA) diet in the summer to a low PUFA diet in the fall before entering torpor [37]. Shorthand naming of individual fatty acids uses the **n-x y:z** nomenclature where **x** indicates the position of the first double bond from the methyl carbon end (**n**), **y** is the total number of carbons in the acyl chain, and **z** is the number of double bonds. Specific fatty acids such as the n-6 PUFA linoleic acid (n-6 18:2) have been shown to enhance hibernation capacity [35,38]. Similarly, some birds drastically improve their ability for aerobic metabolism by feeding on diets high in long-chain n-3 PUFAs. Semipalmated sandpipers double their body mass just before migrating across the Atlantic Ocean between Canada and Brazil by eating large amounts of n-3 PUFA: from mud shrimps loaded with eicosapentaenoic acid (n-3 20:5) and docosahexaenoic acid (n-3 22:6). This “natural doping” strategy greatly improves the aerobic capacity of the long-distance migrant [39,40], and has been further demonstrated experimentally in sedentary quails [32]. Therefore, animals can manipulate the lipid composition of their membranes by selecting particular diets to alter basal metabolism or aerobic capacity for successful hibernation or long-distance migration.

### 2.3. Membrane Pacemaker Theory of Metabolism

The membrane pacemaker theory of metabolism stipulates that the fatty acid composition of membrane phospholipids sets the basal metabolic rate of organisms [41]. Its original formulation was based on the combined observations that: (i) the mass-specific metabolic rate of animals decreases with body size, (ii) the average number of double bonds in membrane fatty acids also decreases with size, and (iii) integral proteins are activated when membrane unsaturation increases [41]. This theory was inferred from the correlation between the lipid composition of membranes and body size discovered in mammals [23], and was subsequently supported by many other studies [21,42]. The validity of the pacemaker theory has been questioned, however, because the relationship between body size and membrane composition first characterized in mammals disappears when the effects of phylogeny are taken into account [43]. More recently, analyses using orchid bees [44] and cypriniform fish [45] provide support for the theory, even after correction for phylogeny. If the metabolic rate of organisms is set by membrane composition on an evolutionary time scale, the same mechanism could be used to suppress metabolism in hypoxia within an animal’s lifespan.

### 2.4. Potential Role for Membrane Regulation of ATP Use and Production in Chronic Hypoxia

The main pitfall faced by organisms exposed to prolonged hypoxia is their ultimate failure to match ATP supply with demand [5]. Thus, it is imperative to downregulate ATP-consuming and ATP-supplying processes to survive in low O_2_ environments. This can be achieved by modulating enzymes that play essential roles in regulating ATP use (ATPases) and ATP production (energy metabolism). This section deals with membrane regulation of Na^+^/K^+^-ATPase and key enzymes of energy metabolism during chronic hypoxia. The downregulation of these enzymes via other mechanisms such as epigenetics, post-translational and post-transcriptional modifications as well as AMPK are beyond the scope of this review and are reviewed elsewhere [12,13].

#### 2.4.1. Membrane Regulation of Na^+^/K^+^-ATPase (ATP Use)

Na^+^/K^+^-ATPase is an integral protein that is responsible for approximately 25% of total ATP consumption [46]. This enzyme requires constant ATP supply to maintain transmembrane Na^+^ and K^+^ gradients. When ATP production is reduced under O_2_-limiting conditions, Na^+^/K^+^-ATPase becomes the dominant energy sink of the cell [47]. This pump is particularly important in the brain where it drives action potentials by regulating Na^+^ and K^+^ currents. Any failure of its normal function eventually causes a spike in intracellular calcium that can lead to neuronal death [5]. Surprisingly, only a handful of studies have examined the effects of chronic hypoxia on Na^+^/K^+^-ATPase (see Table 1) because the bulk of previous research has focused on acute hypoxia and anoxia. The hypoxia tolerant species measured to date, as well as the rat, downregulate Na^+^/K^+^-ATPase when exposed to chronic hypoxia. Unfortunately, this limited information does not allow one to determine if tolerant and sensitive species show different Na^+^/K^+^-ATPase responses. Previous studies have mostly examined Na^+^/K^+^-ATPase in the vertebrate brain, and it will be important to characterize the effects of chronic hypoxia on other tissues from both, hypoxia-sensitive and -tolerant animals. The general downregulation of Na^+^/K^+^-ATPase observed to date (see Table 1) suggests that ion channels are also inhibited by prolonged hypoxia to prevent a harmful increase in intracellular calcium.

Current evidence shows that the activity of Na^+^/K^+^-ATPase is affected by the local lipid environment, in particular by the relative abundance of specific fatty acids and cholesterol. Multi-species comparisons within birds or mammals show that Na^+^/K^+^-ATPase activity is positively correlated with membrane n-3 22:6 abundance [52], and a clear functional link between these parameters has been demonstrated with cross-species experiments. Na^+^/K^+^-ATPase taken from an ectotherm is activated when reconstituted in a mammalian membrane that is richer in n-3 22:6, and the reverse experiment has confirmed that 22:6 is an activator of this essential pump [53,54]. Finally, changing intrinsic cholesterol abundance downregulates Na^+^/K^+^-ATPase in humans, rabbits, guinea pigs and rats [16,17,18,55].

#### 2.4.2. Membrane Regulation of Glycolysis, β-Oxidation and the Tricarboxylic Acid (TCA) Cycle (ATP Production)

The overall effects of chronic hypoxia on all glycolytic enzymes are variable, but some trends can be observed in endotherms (Table 2, Table 3 and Table 4). No pattern can be found for the glycolytic response of ectotherms, and it is presently unclear whether a general response to chronic hypoxia exists for this group of animals. Endotherms generally upregulate hexokinase (HK; Table 2), maintain phosphofructokinase (PFK; Table 3), and downregulate pyruvate kinase (PK; Table 4). The opposite responses shown by HK (activation) and PK (inhibition) also support the notion that hypoxic endotherms simply maintain normal glycolytic supply of ATP. As the last enzyme of the glycolytic pathway, lactate dehydrogenase (LDH) activity is often used as an indicator of tissue capacity for anaerobic ATP production. The lack of a clear activation or downregulation of LDH by chronic hypoxia in ecto- and endotherms (Table 5) indicates that animals do not generally rely on anaerobic metabolism to survive in hypoxic environments. Glycolytic supply of pyruvate to the TCA cycle seems to be regulated differently among species, irrespective of their tolerance to hypoxia, and this regulation favors metabolic suppression in some cases.

β-oxidation is a mitochondrial pathway that breaks down fatty acids to acetyl-CoA and fuels the TCA cycle. The transmembrane enzyme carnitine palmitoyl transferase (CPT) exerts the strongest control on β-oxidation flux [72], and it is modulated by changes in membrane composition. Chronic hypoxia causes a general decrease in CPT (Table 6) and 3-hydroxyacyl-CoA dehydrogenase (HOAD) activity (another enzyme that regulates β-oxidation; Table 7). The only two exceptions to this pattern are CPT activation in tench liver and red muscle [62], and HOAD activation in goldfish brain [51]. Otherwise, general downregulation of β-oxidation appears to be a common way to adjust ATP supply to the lower energy demand afforded by hypometabolism.

The TCA cycle generates NADH and FADH_2,_ which feed into mitochondria to yield high amounts of ATP. Reliance on this pathway becomes difficult when O_2_ availability is reduced. Most animals respond to chronic hypoxia by downregulating citrate synthase (CS) in various tissues (Table 8). The only two studies showing CS activation are for the heart in sablefish [78] and NMR [15]. Overall, however, flux capacity through the TCA cycle is lowered in animals exposed to chronic hypoxia. The general decrease in CS activity also indicates that mitochondrial density is reduced across tissues [71]. 

Specific membrane fatty acids influence the activities of enzymes involved in energy metabolism. Briefly, fast glycolytic muscles contain more palmitic acid (16:0) and n-6 18:2, but less long-chain PUFA [79] than slow oxidative muscles, suggesting that glycolytic enzymes are downregulated by long-chain PUFA. Moreover, n-3 PUFA levels are positively correlated with the activities of β-oxidation and TCA cycle enzymes [80]. This is clearly evident in sedentary quails [32] and migrant sandpipers [40] that activate CS, HOAD and CPT after incorporating dietary long-chain n-3 PUFA in their membranes. The activity of CPT also increases in the presence of more n-3 20:5 in the membranes of adipocytes [81]. No information is currently available on whether modulating membrane cholesterol has similar repercussions on energy metabolism pathways as it does on ATPases. Overall, however, there is strong evidence that altering membrane phospholipids and cholesterol affects ATP supply and demand by activating or inhibiting key enzymes. 

### 2.5. Evidence for Membrane Regulation of Ion Channels

Reducing ion pump activity in hypoxia is only possible with a matching decrease in ion leak so that vital transmembrane ion gradients are preserved. Therefore, it is essential to examine whether ion channels can also be downregulated by changing the lipid composition of membranes. Ion channels are integral membrane proteins surrounded by lipids and they contain a transmembrane domain that moves within the bilayer [82]. Membrane lipids are known to modulate ion channels directly or indirectly via specific lipid–protein interactions. This section reviews known mechanisms whereby changes in membrane PUFAs and cholesterol alter the function of Ca^2+^, K^+^ and Na^+^ channels as well as nicotinicoid receptors [82,83]. Depleting cholesterol experimentally causes an increase in Ca^2+^ uptake through the Ca^2+^ channel and the Na^+^/Ca^2+^ exchanger of the sarcolemma, whereas cholesterol enrichment decreases conductance of the Ca^2+^-dependent K^+^ channel [19,82]. Negatively charged long chain fatty acids upregulate Ca^2+^-activated K^+^ channels with the strongest effect observed for cis unsaturated fatty acids [83]. Modifying membrane cholesterol from normal levels reduces the activity of K^+^ channels [83,84]. Moreover, voltage-gated Na^+^ channels are inhibited by PUFAs [85,86] and cholesterol [84]. This occurs by shifting the steady-state inactivation kinetics of this voltage-gated ion channel in the direction of hyperpolarization, possibly via selective binding to the inactive site of the channel [86].

Membrane lipids also impact the function of ligand-gated ion channels such as nicotinicoid receptors. They include the excitatory acetylcholine and serotonin receptors as well as the inhibitory gamma-aminobutyric acid (GABA) receptors that are required to propagate neuronal signals. Functional acetylcholine receptors depend on the presence of both cholesterol and negatively charged phospholipids to support ion flux. On its own, cholesterol also alters the function of GABA, serotonin and acetylcholine receptors because this sterol is necessary for maintaining their optimal activity [82,83]. It has also been suggested that a decrease in hippocampal cholesterol levels could reduce N-methyl-D-aspartate receptor (NMDA) signaling [87]. Overall, current information clearly shows that membrane lipids do not only modulate ion pumps, but also ion channels.

### 2.6. Potential Role of Membranes in the Regulation of Mitochondrial Function

Mitochondria are major O_2_ consumers that produce ATP through oxidative phosphorylation: a process that couples the electron transport chain (ETC = enzyme complexes I to IV) with ATP synthase (complex V). The ETC pumps protons across the inner mitochondrial membrane to establish an electrochemical gradient that is used to phosphorylate ADP [88]. Oxygen is consumed at complex IV (cytochrome c oxidase, COX): the final electron acceptor that reduces O_2_ to water and contributes to generating the proton gradient [89]. All these protein complexes are transmembrane enzymes whose activities are modulated by changes in the phospholipid composition of the bilayer. Mitochondria also produce significant amounts of reactive oxygen species (ROS), particularly at complexes I and III [90]. These organelles are strategically placed to sense any changes in O_2_ and initiate organism-specific responses to hypoxia. Oxygen sensing can be done through a ROS-induced response that may cause rapid accumulation of Ca^2+^ and/or activation of hypoxia inducible factor (HIF) [88]. ROS can cause the formation of disulfide bonds, which may change the structure and function of proteins such as phosphatases, transcription factors and those involved in epigenetic modifications [12]. Severe hypoxia causes the depolarization of mitochondria that leads complex V to switch from ATP production to ATP consumption [91]. This exacerbates the existing ATP shortfall already induced by hypoxia and can eventually result in tissue failure. This section deals with the effects of chronic hypoxia and membrane lipids on the functional capacity of mitochondria by examining specific responses for the different respiration states and ETC complexes.

The effects of chronic hypoxia on the mitochondria of endotherms have only been investigated in rats and deer mice. After acclimation to hypoxia, rats decrease respiration capacity through ETC complexes I, II and IV in the heart [92] as well as state 3 (OXPHOS in the presence of substrates and ADP) and 4 (LEAK after ADP depletion) in the brain [93]. However, this response is not always consistent because another study shows no change in rat liver and heart [94]. Hypoxia-tolerant species like deer mice have different mitochondrial responses to low O_2_ to rats. They increase mitochondrial respiration (complexes I, II and IV) in the diaphragm [95], but maintain it in the gastrocnemius. In addition, mitochondrial respiration capacity is higher in high-altitude vs. lowland deer mice [96].

Exposing ectotherms to prolonged hypoxia results in a wide-range of mitochondrial responses that do not follow a general trend. For instance, mitochondrial respiration for various states and tissues is lowered in frogs [97] and eastern oysters [98], but remains unchanged in killifish liver [99] and snapper heart [100]. Respiration states of goldfish mitochondria show different responses across tissues [51]. Overall, it is impossible to predict how mitochondria respond to chronic hypoxia because some animals maintain respiration capacity while many others prefer to: (i) regulate specific respiration states that impact ATP turnover, (ii) change mitochondrial efficiency, (iii) change mitochondrial abundance, or (iv) use a combination of the above.

The known effects of chronic hypoxia on the lipid composition of the bilayer do not deal specifically with mitochondria, but with total tissue membranes [14,15]. Because mitochondrial membranes can sometimes respond to stress differently than other membranes (plasma, sarcolemma, endoplasmic reticulum), it would be interesting to investigate whether/how hypoxia affects isolated mitochondrial membranes. Diet-induced changes in membrane lipids are known to alter mitochondrial function in fish [31,101] and hibernating mammals [102]. For example, rainbow trout fed a n-3 22:6-rich diet increase state 3 [101] and state 4 respiration [31]. Early studies also show that the fatty acid composition of the diet has an impact on the hibernation capacity of golden-mantled ground squirrels [103,104]. This interesting observation has been further explored recently in an attempt to characterize physiological links between membrane composition, mitochondrial function, hibernation capacity, and metabolic suppression. For instance, ground squirrel mitochondria suppress state 3 respiration during torpor, especially when using succinate as a substrate [105]. Changing the levels of n-6 18:2 in the diet of 13-lined ground squirrels can also markedly reduce state 3 respiration or proton leak [106], and these reductions could be used to conserve energy in hibernation [102]. The authors suggest that changes in membrane lipid composition could help to reduce the respiration capacity of mitochondria during torpor, although other mechanisms are also clearly involved [107]. Cardiolipin is a major phospholipid constituent of the inner mitochondrial membrane and plays a role in regulating complexes I [108], IV (COX) [109] and V [110]. Thus, it would be particularly useful to find out whether chronic hypoxia affects cardiolipin abundance in mitochondrial membranes. In summary, the remodeling of membrane lipids can clearly modulate mitochondrial respiration capacity and this mechanism could be used to promote metabolic suppression.

### 2.7. Potential Role of Membrane Lipids in Supporting Metabolic Suppression during Chronic Hypoxia

**Figure 1:**
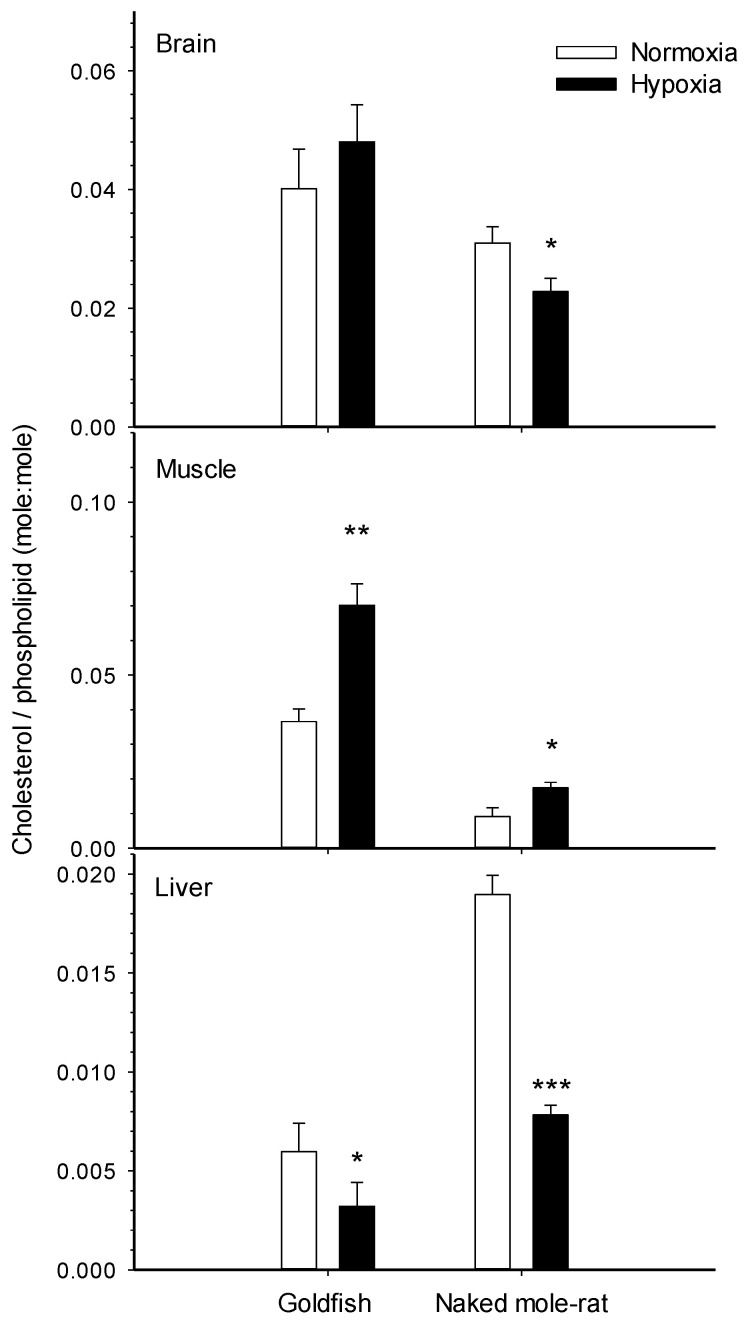
Relative membrane cholesterol in the tissues of normoxic controls and hypoxia-acclimated animals for two hypoxia-tolerant vertebrates: the goldfish [14] and the naked-mole rat [15]. Values are means ± SEM (*n* = 9–16 per treatment). * *p* < 0.05, ** *p* < 0.01, *** *p* < 0.001 indicate significant effects of hypoxia.

**Figure 2:**
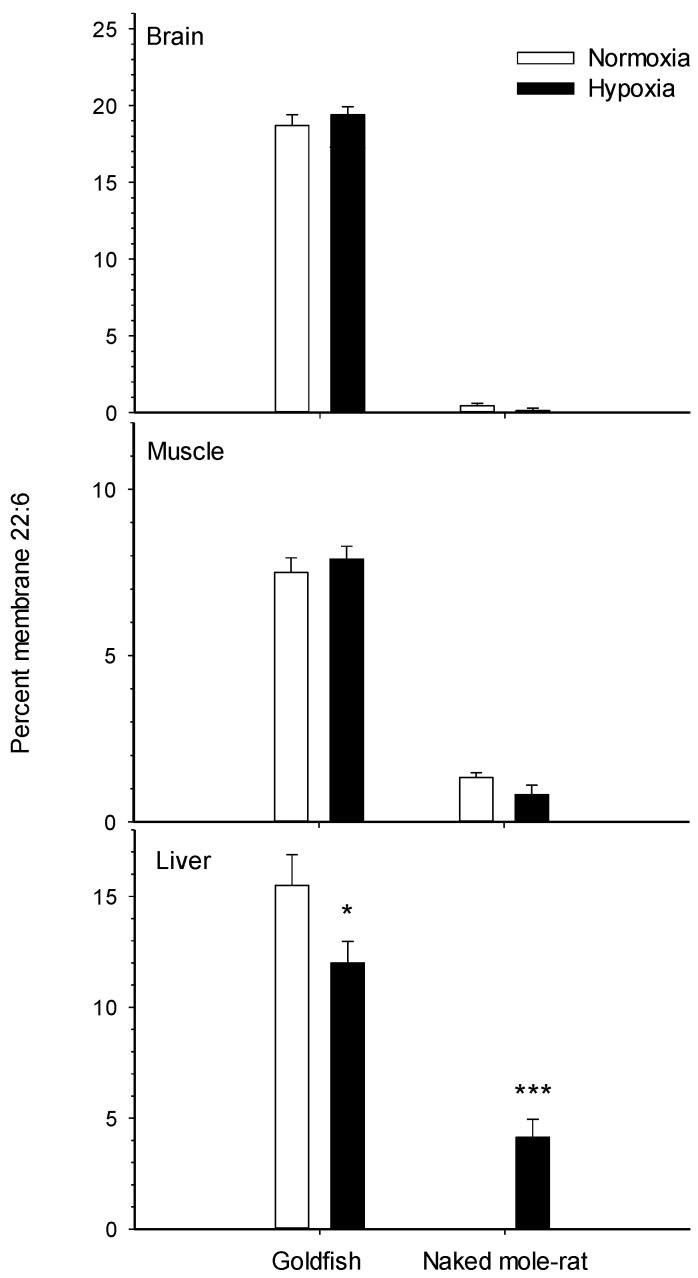
Percent docosahexaenoic acid (n-3 22:6) in membrane phospholipids in the tissues of normoxic controls and hypoxia-acclimated animals for two hypoxia-tolerant vertebrates: the goldfish [14] and the naked-mole rat [15]. Values are means ± SEM (*n* = 9–14 per treatment). * *p* < 0.05, *** *p* < 0.001 indicate significant effects of hypoxia.

The membrane restructuring effects of temperature, toxins and diet discussed in Section 2.1 and Section 2.2 suggest that other environmental factors like hypoxia could also influence lipid composition. Recent experimental evidence demonstrates that this is the case in two hypoxia tolerant vertebrates. We have shown that goldfish [14] and NMRs [15] undergo widespread remodeling of membrane lipids in response to prolonged in vivo exposure to low O_2_. Both species modulate cholesterol extensively (Figure 1), but the effects of chronic hypoxia on membrane phospholipids are more pronounced in goldfish [14] than NMRs [15] (Figure 2). Changing cholesterol abundance strongly affects the activity of membrane proteins [16,17,18,19,20]. Goldfish and NMR increase cholesterol in muscle and decrease it in liver, but only NMRs show a decrease in the brain. These responses are intriguing because multiple studies on artificial membranes [16,17] and manipulated fish membranes [18] show that changing cholesterol levels generally downregulates Na^+^/K^+^-ATPase. Therefore, this hypoxia-driven membrane response could contribute to metabolic suppression by lowering ATP demand. Another way to reduce Na^+^/K^+^-ATPase activity is to decrease the abundance of n-3 22:6 in membrane phospholipids because this fatty acid is an activator of the ion pump [21,22]. Lowering n-3 22:6 levels also suggests a downregulation of ATP supply from β-oxidation and the TCA cycle because n-3 PUFA are positively correlated with the maximal enzymatic activities of CPT, HOAD and CS [33,40,41,62]. Such a strategy for lowering ATP demand and supply seems to be used by the goldfish that reduces %22:6 of liver (Figure 2) and gill membranes [14]. However, this mechanism is not available to NMRs because of their intrinsically low levels of n-3 22:6 (only 0–4% of total membrane fatty acids in normoxic animals) that leaves little to no room for a decrease during hypoxia [15]. Overall, the hypoxia-driven changes in membrane lipids observed to date could represent a novel mechanism to reduce organismal metabolic rate in stressful environments. It is presently unclear whether this membrane response to chronic hypoxia only occurs in hypoxia tolerant species or more generally in all animals.

## 3. Conclusions and Future Directions

**Figure 3:**
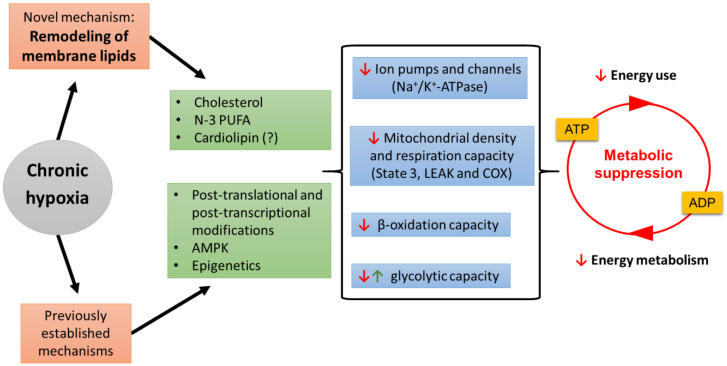
Remodeling of membrane lipids is a proposed new mechanism to promote metabolic suppression in chronic hypoxia. Prolonged in vivo exposure to low oxygen alters the relative abundance of membrane cholesterol, n-3 polyunsaturated fatty acids (PUFA), and possibly mitochondrial cardiolipin in ways that downregulate ion pumps such as Na^+^/K^+^-ATPase (a major ATP consumer), ion channels, and possibly mitochondrial respiration capacity [state 3 (OXPHOS in the presence of substrates and ADP) and LEAK (proton leak)]. Chronic hypoxia also causes a general reduction in cytochrome c oxidase (COX) indicating a decrease in mitochondrial density. The observed changes in membrane composition are known to modulate metabolic pathways of energy metabolism such as β-oxidation (downregulation) and glycolysis (up or downregulation). Reduction of flux through the ATP-ADP cycle can also be induced by previously characterized mechanisms that involve post-translational/post-transcriptional modifications [11], 5′-AMP-activated protein kinase (AMPK) [12] or epigenetic changes [13]. Membrane remodeling and established mechanisms work in concert to cause metabolic suppression.

Current understanding of the mechanisms that cause metabolic suppression in chronic hypoxia are summarized in Figure 3. The lipid composition of membranes plays a fundamental role in setting the metabolic capacity of cells, tissues and organisms. This review has examined how animals use membrane plasticity to adjust their metabolic capacity in a wide range of physiological situations and it proposes that chronic hypoxia is among them. This versatile mechanism is maybe best exemplified by birds that boost aerobic capacity before long-distance migration (natural doping in sandpipers), scaling of mass-specific metabolic rate (membrane pacemaker theory of metabolism), and various studies of metabolic suppression (hibernator mitochondria and downregulation of Na^+^/K^+^- ATPase in artificial membranes).

A successful hypometabolic response relies primarily on reducing ATP use whereas lowering ATP production is easier to achieve and can be done secondarily. Therefore, mechanisms that downregulate ion pumps, ion channels and proton leak play a strategic role in setting the tolerance of organisms for hypoxia. Recent studies suggest that hypoxia-induced suppression of metabolism is partly mediated by membrane plasticity through multiple mechanisms. Organismal acclimation to low oxygen causes widespread changes in the lipid composition of membranes in two champions of hypoxia tolerance: the goldfish and the naked mole-rat (see Section 2.7). To cope with this environmental stress, these resilient animals modify their membranes in ways that support the downregulation of key enzymes directly involved in ATP turnover. In hypoxia tolerant species, entering the hypometabolic state occurs together with extensive changes in membrane cholesterol, a decrease in mitochondrial density, as well as the downregulation of brain Na^+^/K^+^-ATPase and acetyl-CoA supply from β-oxidation and/or glycolysis. However, a direct functional link between changes in membrane lipids and the downregulation of major ATP consuming processes remains to be further established. A common membrane signal regulating the joint inhibition of ion pumps and channels could be an exquisite way to preserve the balance between ATP supply and demand in the hypometabolic state. To investigate how membrane restructuring and metabolic suppression can be mechanistically linked, it will be useful to mimic the membrane changes observed in vivo on artificial membranes to characterize how ion pumps and channels are affected by hypoxia.

Hibernators seem to rely on changes in mitochondrial membrane lipids to reduce proton leak and state 3 respiration as they enter torpor, and the same mechanism could be used in hypoxia (see Section 2.6). Examining the effects of chronic hypoxia on the lipid composition of mitochondrial membranes is therefore an important avenue for future research. Do the hypoxia-driven changes already characterized for total tissue membranes echo those of mitochondrial membranes specifically? The membrane remodeling mechanisms presently linked to metabolic suppression include changes in cholesterol, fatty acid composition of phospholipids, and mitochondrial cardiolipin, but their relative importance is unknown.

While not as critical as reducing ATP use, lowering the rate of ATP synthesis is also required to keep matching ATP supply with demand in hypometabolic states. Maximal flux capacities of key metabolic pathways such as glycolysis, β-oxidation and the TCA cycle are affected by both chronic hypoxia and changes in membrane lipids. To what extent the downregulation of energy metabolism caused by hypoxia depends on membrane remodeling is currently unclear. In conclusion, we propose that membrane restructuring is a novel physiological mechanism used by animals to suppress ATP turnover during prolonged hypoxia.

## Figures and Tables

**Table 1:** Effects of chronic hypoxia (minimum 1 week) on the maximal activity of Na+/K+-ATPase in various animals.

Species	Tissue	Na^+^/K^+^-ATPase Response	Hypoxia Duration (Weeks)	Reference
Mouse (*Mus musculus*)	Brain	-	3	[48]
Rat (*Rattus norvegicus*)	Brain	~30–40% ↓	4	[49]
Naked mole rat (*Heterocephalus glaber*)	Brain	77% ↓	4	[15]
	Liver	41% ↑		[15]
	Temporalis, heart	-		[15]
Crucian carp (*Carassius carassius*)	Heart	33% ↓	3	[50]
Goldfish (*Carassius auratus*)	Brain	40% ↓	4	[51]
	Liver, white muscle	-		[51]

**Table 2:** Effects of chronic hypoxia (minimum 1 week) on the maximal activity of hexokinase (HK) in various animals.

Species	Tissue	HK Response	Hypoxia Duration (Weeks)	Reference
Deer mouse (*Peromyscus maniculatus*)	Gastrocnemius	35% ↑	6–8	[56]
	Gastrocnemius	-		[56]
Mouse (*Mus musculus*)	Brain	-	3	[48]
Rat (*Rattus norvegicus*)	Gastrocnemius, soleus, heart, brain	8–105% ↑	3–10	[57,58,59,60]
Gulf killifish (*Fundulus grandis*)	Heart, brain	16–28% ↑	4	[9]
	Liver	-		[9]
Goldfish (*Carassius auratus*)	Brain	12% ↓	4	[51]
	White muscle	82% ↑		
	White muscle, red muscle, liver	-	4–6	[51,61]
Tench (*Tinca tinca*)	White muscle	67% ↓	6	[62]
	Red muscle, liver	-		[62]
Chinese shrimp (*Fenneropenaeus chinensis*)	pancreas, pleopod, abdominal muscle	24–26% ↓	2	[63]

**Table 3:** Effects of chronic hypoxia (minimum 1 week) on the maximal activity of phosphofructokinase (PFK) in various animals.

Species	Tissue	PFK Response	Hypoxia Duration (Weeks)	Reference
Deer mouse (*Peromyscus maniculatus*)	Gastrocnemius	-	6–8	[56]
Rat (*Rattus norvegicus*)	Heart, soleus, gastrocnemius, caudal nerve	-	3–10	[57,58,59]
Gulf killifish (*Fundulus grandis*)	White muscle	25% ↓	4	[9]
	Liver	63% ↑		[9]
	Heart, brain	-		[9]
Nile tilapia (*Oreochromis niloticus*)	Liver, white muscle	59–123 ↑	4	[64]
Tench (*Tinca tinca*)	White muscle	-	6	[62]
	Red muscle, liver	98–120% ↑		[62]
Chinese shrimp (*Fenneropenaeus chinensis*)	hepatopancreas, pleopod, abdominal muscle	16–31% ↓	2	[63]

**Table 4:** Effects of chronic hypoxia (minimum 1 week) on the maximal activity of pyruvate kinase (PK) in various animals.

Species	Tissue	PK Response	Hypoxia Duration (Weeks)	Reference
Deer mouse (*Peromyscus maniculatus*)	Gastrocnemius	-	6–8	[56]
Rat (*Rattus norvegicus*)	Heart, soleus	-	3	[58]
	Gastrocnemius	-	4	[57]
Naked mole-rat (*Heterocephalus glaber*)	Liver, temporalis muscle, brain, heart, kidney	61–99% ↓	4	[15]
Mouse (*Mus musculus*)	Liver	65% ↓	4	[65]
Northern shrimp (*Pandalus borealis*)	White muscle	-	1	[66]
Greenland halibut (*Reinhardtius hippoglossoides*)	White muscle	46% ↓		[66]
Common carp (*Cyprinus carpio*)	White muscle	-	1	[67]
Nile tilapia (*Oreochromis niloticus*)	Liver	61–96% ↑	4	[64]
	White muscle	-		[64]
Gulf killifish (*Fundulus grandis*)	White muscle	23% ↓	4	[9]
	Heart	24% ↑		[9]
	Liver, brain	-		[9]
Goldfish (*Carassius auratus*)	White and red muscle, liver	-	6	[61]
	Liver	47% ↓	4	[51]
	White muscle, brain	-		[51]
Tench (*Tinca tinca*)	White and red muscle	-	6	[62]
	Liver	86% ↑		[62]
Chinese shrimp (*Fenneropenaeus chinensis*)	hepatopancreas, pleopod, abdominal muscle	14–39% ↓	2	[63]

**Table 5:** Effects of chronic hypoxia (minimum 1 week) on the maximal activity of lactate dehydrogenase (LDH) in various animals.

Species	Tissue	LDH Response	Hypoxia Duration (Weeks)	Reference
Deer mouse (*Peromyscus maniculatus*)	Gastrocnemius, diaphragm	-	6–8	[68,69]
Mouse (*Mus musculus*)	Hindlimb muscles	28% ↓	1	[70]
	Brain, liver	-	3–4	[48,65]
Rat (*Rattus norvegicus*)	Soleus	-	3	[58]
	Gastrocnemius, heart, gastrocnemius and liver mitochondria	25–54% ↑	1–3	[65,66,71]
Naked mole-rat (*Heterocephalus glaber*)	Brain, liver, temporalis	62–82% ↓	4	[15]
	Kidney	81% ↑		[15]
	Heart	-		[15]
Northern Shrimp (*Pandalus borealis*)	White muscle	45–88% ↓	1	[66]
Greenland halibut (*Reinhardtius hippoglossoides*)	White muscle	58% ↓		[66]
Common carp (*Cyprinus carpio*)	White muscle	-	1	[67]
	Liver	~60% ↑		[67]
Nile tilapia (*Oreochromis niloticus*)	Liver, white muscle	80–176% ↑	4	[64]
Gulf killifish (*Fundulus grandis*)	White muscle	30% ↓	4	[9]
	Liver	30% ↑		[9]
	Heart, brain	-		[9]
Goldfish (*Carassius auratus*)	White muscle, red muscle, liver, brain	-	4–6	[51,61]
Tench (*Tinca tinca*)	White and red muscles	-	6	[62]
	Liver	116% ↑		[62]
Chinese shrimp (*Fenneropenaeus chinensis*)	hepatopancreas, pleopod, abdominal muscle	26–33% ↓	2	[63]

**Table 6:** Effects of chronic hypoxia (minimum 1 week) on the maximal activity of carnitine palmitoyl transferase (CPT) in various animals.

Species	Tissue	CPT Response	Hypoxia Duration (Weeks)	Reference
Rat (*Rattus norvegicus*)	Muscle, heart	16–34% ↓	5	[73,74]
	Liver, gastrocnemius mitochondria	-	1–5	[74,75]
Naked mole-rat (*Heterocephalus glaber*)	Liver, temporalis muscle	89–98% ↓	4	[15]
	Brain, heart, kidney	-		[15]
Mouse (*Mus musculus*)	Skeletal muscle	65% ↓	1	[76]
	Heart	-		[76]
Goldfish (*Carassius auratus*)	Brain	18% ↓	4	[51]
	Liver, white muscle	-		[51]
Tench (*Tinca tinca*)	Red muscle, liver	162–236% ↑	6	[62]
	White muscle	-		[62]

**Table 7:** Effects of chronic hypoxia (minimum 1 week) on the maximal activity of 3-hydroxyacyl-CoA dehydrogenase (HOAD) in various animals.

Species	Tissue	HOAD Response	Hypoxia Duration (Weeks)	Reference
Deer mouse (*Peromyscus maniculatus*)	Gastrocnemius, liver	-	6–8	[56]
Mouse (*Mus musculus*)	Left ventricle	36% ↓	4	[77]
Rat (*Rattus norvegicus*)	Heart, skeletal, liver and liver mitochondria	20–71% ↓	1–5	[58,73,74,75]
	Soleus, gastrocnemius mitochondria	-	1–3	[58,75]
Naked mole-rat (*Heterocephalus glaber*)	Liver, temporalis muscle	69–93% ↓	4	[15]
	Brain, heart, kidney	-		[15]
Mouse (*Mus musculus*)	Heart, skeletal	-	1	[76]
Goldfish (*Carassius auratus*)	Brain	70% ↑	4	[51]
	Liver, White muscle	-		[51]

**Table 8:** Effects of chronic hypoxia (minimum 1 week) on the maximal activity of citrate synthase (CS) in various animals.

Species	Tissue	CS Response	Hypoxia Duration (Weeks)	Reference
Deer mouse (*Peromyscus maniculatus*)	Liver, gastrocnemius, diaphragm	-	6–8	[64,76,77]
Mouse (*Mus musculus*)	Liver mitochondria	34% ↓	1	[75]
	Hindlimb muscles, heart	-	1–4	[70,77]
	Gastrocnemius mitochondria	-	1	[75]
	Brain, liver	-	3–4	[48,65]
Rat (*Rattus norvegicus*)	Gastrocnemius	34–39% ↓	4	[57]
	Gastrocnemius, heart, liver	-	3–5	[66,79,80]
Naked mole-rat (*Heterocephalus glaber*)	Brain, liver, temporalis, kidney	25–78% ↓	4	[15]
	Heart	94–115% ↑		[15]
Goldfish (*Carassius auratus*)	Brain, liver, white muscle	-	4	[51]
Common carp (*Cyprinus carpio*)	White muscle	~25% ↓	1	[67]
	Liver	-		[67]
Northern shrimp (*Pandalus borealis*)	White muscle	40% ↓	1	[66]
Greenland halibut (*Reinhardtius hippoglossoides*)	White muscle	33% ↓		[66]
Chinese shrimp (*Fenneropenaeus chinensis*)	pancreas, pleopod, abdominal muscle	31–70% ↓	2	[63]
Sablefish (*Anoplopoma fimbria*)	Heart	20% ↑	3	[78]
